# From point sources to phantoms: refining dose rate models in nuclear medicine with Tc-99m, F-18, I-131 and Lu-177

**DOI:** 10.1007/s13246-026-01775-7

**Published:** 2026-07-23

**Authors:** Joshua Ierace, Pejman Rowshanfarzad, Christine Sinnott, Rikki Nezich, Paul Brayshaw, Mario Djukelic

**Affiliations:** 1 School of Physics, Mathematics and Computing, University of Western Australia, Crawley, WA Australia; 2 Centre for Advanced Technologies in Cancer Research (CATCR), Perth, WA Australia; 3 Department of Medical Technology and Physics, Sir Charles Gairdner Hospital, Nedlands, WA Australia; 4 Department of Nuclear Medicine and WA PET Centre, Sir Charles Gairdner Hospital, Nedlands, WA Australia; 5 GenesisCare WA, 24 Salvado Rd, Wembley, WA 6014 Australia

**Keywords:** Nuclear medicine, Dose rate measurement, Gamma factor, Self-absorption factor, Radiation protection, Theranostics

## Abstract

Healthcare workers are often exposed to external radiation from patients who have been administered radionuclides for nuclear medicine (NM) studies, particularly during subsequent procedures requiring close contact. Traditional dose rate estimates commonly assume patients to be unattenuated point sources and apply inverse square law calculations, which tend to overestimate exposures at clinical distances. This study presents an experimental approach to quantify dose rates more accurately from NM patients using point, line, and phantom source models of Tc-99m, F-18, I-131, and Lu-177. Measurements were performed with calibrated ionisation chambers under controlled low-background conditions. Radionuclide activities were selected to achieve detectable dose rates while minimising staff exposure. Point and line source measurements provided baseline external exposure data across 10–100 cm. Phantom models, including a homogeneous water-filled NEMA body phantom and a PMMA neck phantom, were used to replicate clinical patient geometries. Dose rates were decay-corrected, normalised to activity, and fitted with biexponential models to generate exposure curves. Gamma factors (GFs) were evaluated at 0.3 m and 1.0 m from the centre of the radioactive source and compared with internationally recognised literature values. Self-absorption factors (SFs) were derived by comparing phantom and point source curves across clinical distances. Measured GFs showed excellent agreement with published data and internationally recognised standards, differing by only 2% for F-18 at both 30 cm (1.84 μSv/h/MBq) and 100 cm (0.161 μSv/h/MBq), and by 1% for I-131 at 30 cm (0.721 μSv/h/MBq). The average SF for F-18 (0.71 ± 0.04), representing patient self-attenuation for an unknown body orientation, was consistent with published Monte Carlo estimates. Differences reported in the literature were attributed to methodological variations, including the use of computational modelling rather than direct experimental measurements, along with variations in phantom design, source geometry, and detector configuration. This work presents an experimentally measured dataset with corresponding dose rate models for NM applications. The results contribute additional empirical evidence that may be used alongside established radiation protection methodologies to support assessments of shielding, procedure scheduling, and occupational and public radiation risk, potentially reducing reliance on overly conservative assumptions when evaluating dose rates at clinical distances.

## Introduction

Healthcare workers and other allied health professionals have demonstrated concerns regarding external exposure from the use of radionuclides during nuclear medicine (NM) procedures [1, 2]. Occasionally, an NM patient may require a consultation, ultrasound, or surgery after being administered radiopharmaceuticals, while still radioactive. The staff member performing the procedure will be in close proximity to the patient and therefore exposed to higher levels of ionising radiation. It is therefore necessary to quantitatively estimate the radiation dose that healthcare workers may receive during such procedures, at specified distances, for a range of radionuclides commonly used in NM.

NM scans are taken via the emission of photons from inside the patient, where the body metabolises the radiopharmaceutical and the uptake within the body is measured with an external detector. The captured images from NM hybrid imaging modalities will display which organs uptake the radiopharmaceutical and provide physiological information for organs and tissues in the patient’s body along with anatomical information from the low dose computed tomography (CT) or magnetic resonance imaging (MRI). This process allows visualisation and tracking of organ function in order to diagnose disease, leading to early cancer detection. Single-photon emission computed tomography (SPECT) and positron emission tomography (PET) are the primary imaging modalities, most commonly employing Tc-99m and F-18, respectively. Radionuclides are also often used to deliver therapy to patients with various types of cancer and disease. With the rising adoption of theranostics, particularly Lu-177 in peptide receptor radionuclide therapy for neuroendocrine tumours and prostate-specific membrane antigen radiopharmaceutical therapy for prostate cancer [3–6], healthcare providers will come into close contact with patients needed treatment more frequently, increasing their radiation exposure burden.

When calculating the absorbed dose to an individual from radioactive patients, the activity distribution is usually assumed to be an unattenuated point source, as outlined in US-NRC Regulatory Guide 8.39 [7], and the dose to exposed individuals at a given distance is typically estimated using the inverse square law [8]. Considering an NM patient as a point source is simple but overly conservative [9–11]. The US-NRC Regulatory Guide 8.39 [7] incorporates conservatism when calculating the maximum dose rate to members of the public by failing to consider patient body tissue attenuation as well as time-dependent redistribution and excretion of the administered radiopharmaceutical [12]. Both Willegaignon et al. [13] and Yi et al. [2] found that, regardless of the source model applied, the calculated dose rates generally exceeded measured results from patients. They therefore suggested “correction factors” or “self-shielding factors” to reduce overestimation and improve the accuracy of dose estimates [9].

Siegel et al. [8] proposed the line source model to compute exposure rates more accurately, particularly at closer distances. Broggio [10] further examined the robustness of the patient-as-line-source model and concluded that it is a reasonable approximation for Tc-99m, F-18, and I-131. Most patients receiving radiopharmaceuticals, however, are more similar to volume sources than to point or line sources, as much of the administered activity is quickly dispersed in the blood [9]. Young [14] performed dose rate measurements using phantoms for five different radionuclides commonly used in NM departments, including, Tc-99m, F-18, and I-131. The study demonstrated that applying the inverse square law leads to overestimation of dose rates, particularly at short distances. This occurs because the inverse square law assumes an idealised point source, ignoring the physical dispersion of the source and the attenuation effects within the source. Studies [8, 14] have highlighted the limitations of using the inverse square law and unattenuated point source models for representing NM patients.

There is considerable variability across the literature regarding published values of specific gamma exposure constants and self-shielding factors for different radionuclides, arising from differences in the methods used to calculate, estimate, or experimentally determine these quantities. Published values are typically determined at 0.3 m and/or 1.0 m, with dose rates at other distances commonly extrapolated using the inverse square law. This approach does not fully reflect the range of distances encountered by staff when working with NM patients (10–100 cm), and it also neglects consistent source geometry, attenuation within the patient, and detector setup. As a result, there remains value in empirically characterising dose–distance behaviour under controlled, reproducible conditions that mirror clinical practice. The experimentally derived data provided may be compared or integrated with existing phantom-based and Monte Carlo studies that evaluate dose rates as a function of distance, orientation, organ localisation, and body habitus. These geometry-specific reference values are intended to support conservative assessments of external exposure by reducing over-reliance on unattenuated point-source assumptions.

The aim of this study was to systematically characterise dose rate behaviour as a function of distance for radionuclides commonly used in NM facilities by performing measurements using point, line, and phantom source models. Measurements were conducted for Tc-99m, F-18, I-131, and Lu-177 across clinically relevant distances (10–100 cm) using a consistent and robust experimental setup. Geometry-specific dose-distance relationships were derived and compared with the literature to characterise dose rate behaviour across clinically relevant distances. Phantom measurements were also conducted to quantify self-attenuation effects, providing experimentally derived reference values that may complement existing approaches to occupational exposure assessment and shielding calculations through an improved characterisation of dose–distance relationships and self-attenuation effects.

## Materials and methods

**Fig. 1:**
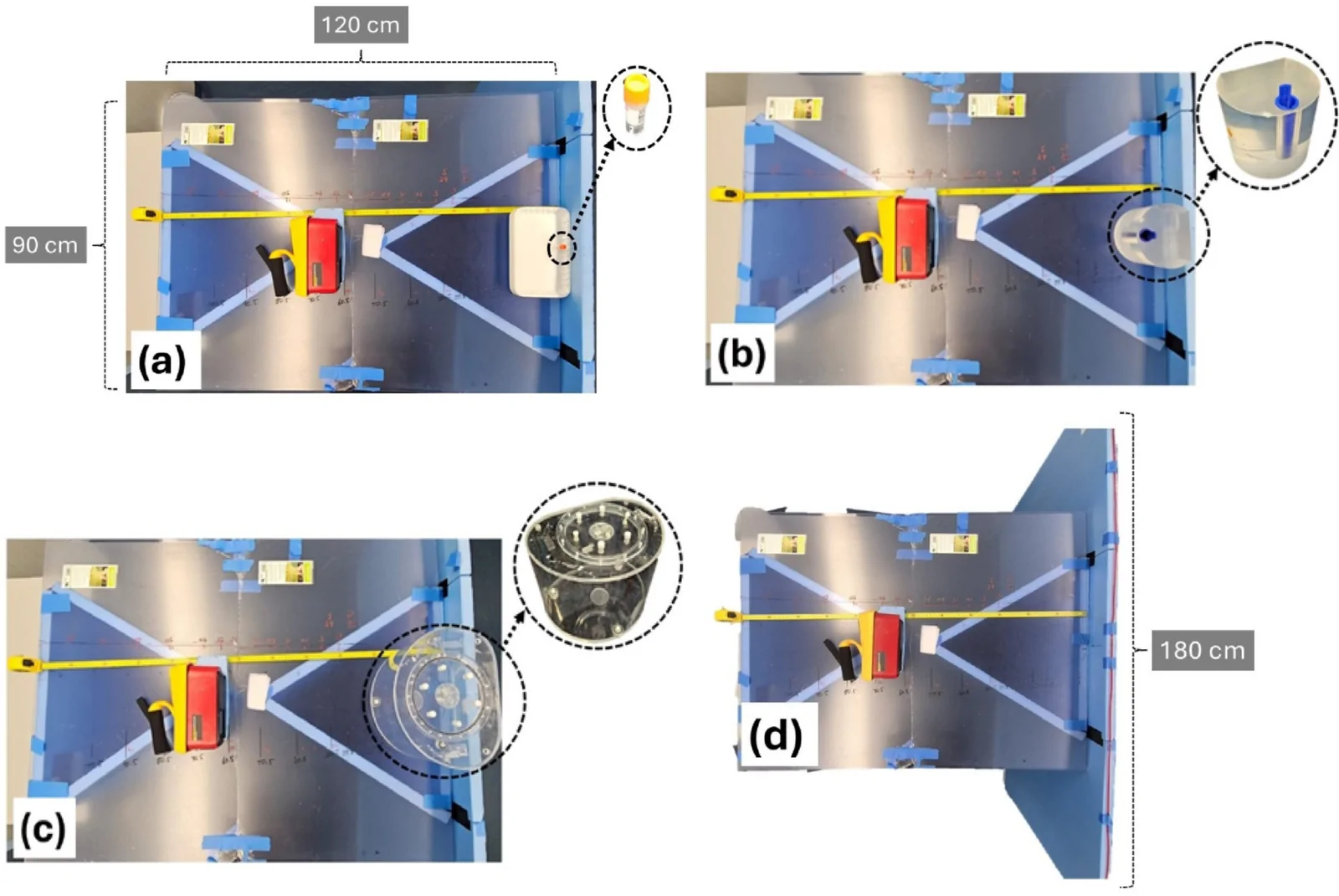
Aerial views of experimental setup for various radioactive sources and models. **a** Setup used for point source model. Vials of radioactive liquid and/or capsule were used for measurement. **b** Setup used for neck phantom source model. A neck phantom with an insert was used for I-131 phantom measurements. The length, width, height, insert diameter and insert depth of the phantom were 12.5 cm, 15.3 cm, 15 cm, 3.3 cm, and 9.9 cm, respectively. **c** Setup used for the NEMA phantom source model. The height, width, and length of the phantom were 22 cm, 23 cm, and 32 cm, respectively. **d** Setup used for line source model. Radioactive material was aspirated through a syringe into a plastic tube and used for measurement

This study was conducted to evaluate dose rate measurements using various radioactive sources and phantoms under controlled experimental conditions. The setup, measurement techniques, and analysis methods are described below. An overview of the experimental configurations used for different source and phantom models is provided in Fig. 1.

In order to conduct this research, physical measurements from different radioactive sources were recorded in an environment with low background radiation levels and minimal backscatter. The measurements took place in the centre of a 3.57 × 3.03 m^2^ outpatient room at the clinic. Three sheets of multi-use foam boards made from extruded polystyrene (Foamex, Australia) were taped firmly together in a triangular configuration, and one sheet of Handi-Screen clear PVC sheet (Suntuf, Australia) was taped on top to create a solid base for the experiments. The top of the base was 120 cm above the floor. Distances used for measuring dose rates were accurately determined and marked on the PVC sheet with a permanent marker. The measurement distances were selected as they represent clinical proximity, that is, distances where staff are interacting with NM patients. The detector was slid across the top sheet, and the dose rate was recorded at each marked distance. The marks directly correspond to the distance between the source or the centre of the phantom to the calibration point on the detector.

### Detector

The dose rates for the measurements described in the following sections were measured using an ionisation chamber survey meter. The model of the ionisation chamber survey meter was the 451B-DE-SI-RYR (Fluke Biomedical, USA). Each ionisation chamber was externally and annually calibrated by Landauer Fluke Biomedical, an institution traceable to primary standards who performed dose rate calibration using three Cs-137 sources with activities of 5 mCi, 50 mCi, and 500 mCi. For the experiments, the detector was secured on a sliding foam to ensure the calibration point was level with the source.

### Activity

The activity in the phantom, line and point source was measured using a well-type ionisation chamber or dose calibrator, CRC-15R, CRC-25R, and CRC-55t (Capintec, USA). The chosen activities for each radionuclide were based on estimations sufficient to generate at least 10 μSv/h at 1 m which ensured a balance of high sensitivity and low external exposure to staff. The radionuclides used in this study included F-18, Tc-99m, I-131, and Lu-177, all of which were in liquid form except for I-131, which was primarily in capsule form. Background radiation levels were measured prior to each measurement and subsequently subtracted from the recorded dose rates.

### Point source

Six measurements were recorded for both F-18 and Tc-99m across three separate days, with activities ranging from 203 to 396 MBq and 913–958 MBq, respectively. The distances for both radionuclides ranged from 11.5 to 96.5 cm, in 5 cm increments up to 26.5 cm, and 10 cm increments thereafter.

Similarly, five measurements, from three separate experiments were recorded for I-131 capsules with activities ranging from 127.5 to 162.7 MBq. Nine measurements were performed for Lu-177, across three experiments, with activities ranging from 1718 to 2147 MBq. The distances for both radionuclides ranged from 13.5 to 98.5 cm, in 5 cm increments up to 28.5 cm, and 10 cm increments thereafter.

Exact times were recorded for each measurement for appropriate decay correction. The vial holding the radionuclide was placed inside a small foam block to ensure it was level with the detector, and to minimise contamination risk.

### Line source

Alterations were made to the setup in preparation for the line source measurements. An additional three foam sheets were taped together, and each sheet was screwed onto the foam base with angle or ‘L’ brackets. The base and the board were made of the same material. The line source was contained within a plastic tube (inner diameter 4.4 mm, outer diameter 5.6 mm) attached to a syringe and placed on top of the three sheets of foam. Each end of the tube was capped and sealed with white tac (UHU, Germany), a plasticine-like material. The line source was securely attached across the top of the foam sheets by tape and 3D printed holders. The line source was positioned 130.7 cm above the floor. Three measurements were recorded on a single day for both F-18 and Tc-99m, with activities of 140.5 MBq and 990 MBq, respectively. The length of the line source for F-18 and Tc-99m was 167 cm and 170 cm, respectively. The distances for both radionuclides ranged from 10 to 110 cm, in 5 cm increments up to 30 cm, and in 10 cm increments thereafter. Dose rates were decay-corrected for the time they were recorded.

### Homogeneous radionuclide and water filled phantom

The selected phantom for the dose rate measurements was the National Electrical Manufacturers Association (NEMA) IEC PET Body Phantom NU 2-2018 model [15]. This phantom was used to represent a patient’s torso, a primary site for radiopharmaceutical accumulation. The height, width and length of the phantom were 22 cm, 23 cm, and 32 cm, respectively. The spheres and lung insert were removed to create a homogeneous phantom. This configuration does not explicitly represent tissue heterogeneities such as bone, lung, or adipose tissue; however, it provides a simplified and reproducible geometry for assessing external dose rate distributions around the torso. Three sets of dose rates were measured for each orientation of the phantom: anterior, posterior, and lateral. In the anterior orientation, distances ranged from 17 to 97 cm, in 5 cm increments up to 27 cm, and in 10 cm increments thereafter. In the posterior orientation, distances ranged from 15 to 95 cm, in 5 cm increments until 25 cm, and in 10 cm increments thereafter. In the lateral orientation, distances ranged from 16.5 to 86.5 cm, in 10 cm increments.

The injected activity of F-18 was 230 MBq. The injected activity of Tc-99m was 1780 MBq and 1300 MBq. The injected activity of Lu-177 was 2600 MBq, and an excess of Diethylenetriamine pentaacetate (DTPA) was added as a chelating agent to prevent the Lu-177 from adsorbing to the phantom walls [6].

### Neck phantom

**Fig. 2:**
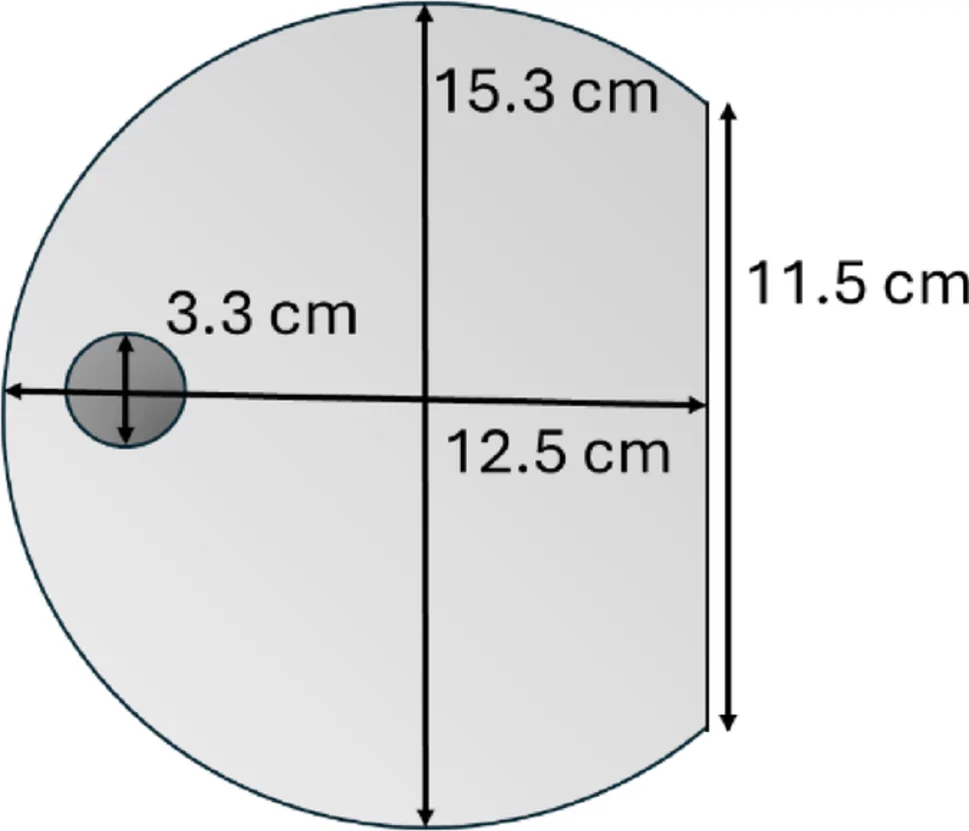
Schematic diagram of Neck phantom used for I-131 phantom source measurements

For the I-131 phantom measurements, an in-house neck phantom made of PMMA was used to simulate an NM patient receiving radioiodine therapy. There was a cut-out in the front of the neck phantom where I-131 could be placed to represent the location where the majority of activity would be present in a patient’s thyroid when receiving treatment. The neck phantom was similar in design to the ANSI/IAEA (American National Standards Institute/International Atomic Energy Agency) neck thyroid phantom [16]. The length, maximum width, height, insert diameter, and insert depth of the phantom were 12.5 cm, 15.3 cm, 15 cm, 3.3 cm and 9.9 cm, respectively, as illustrated in Fig. 2. A 3D-printed hollow dipper, made with 83% PLA, was designed to minimise air attenuation, contamination risk, and handling of the radionuclide. The dipper was created using Fusion 360 (Autodesk, USA) and a Bambu Lab X1C 3D printer (Bambu Lab, China).

Five measurements were recorded across three experiments, with activities ranging from 127.5 to 152.8 MBq. A combination of liquid and capsule samples was used. In the anterior orientation, distances ranged from 6 to 96 cm, in 5 cm increments up to 26 cm, and in 10 cm increments thereafter. In the posterior orientation, distances ranged from 15 to 105 cm, in 5 cm increments up to 35 cm, and 10 cm increments thereafter. In the lateral orientation, distances ranged from 11.5 to 101.5 cm, in 5 cm increments until 31.5 cm, and in 10 cm increments thereafter.

### Data analysis

All data analysis was performed in Microsoft Excel, this includes entering the measured data, decay correcting all dose rates for the time they were recorded and normalising the data to activity (μSv/h/MBq). An average, standard deviation (SD), and standard error (SE) were calculated in Excel. The final results were imported into MATLAB (MathWorks®, USA) to generate plots. The data points for distance and normalised dose rate for all measurements were selected using the curve fitting tool. A non-linear model (NLM) fit with inverse-variance weighting was applied to model the measured data.

The NLM fit was biexponential and followed the structure of Eq. 1, where *x* is the distance from the centre of the source (cm), and F, g, H, and k are fitting parameters. The biexponential model was chosen to describe the measured dose rates, as the observed behaviour reflects the combined influence of scatter and other processes that do not act uniformly with distance. This model incorporates distance-dependent behaviours by using two characteristic components, while maintaining a smooth, monotonic decrease in dose rate across the clinically relevant range. The fitted parameters represent the relative contributions that the exponential variables have on the overall function.

1 $$ y\left( x \right) = Fe^{ - g x} + He^{ - k x} $$

Residual analysis presented in Figures 8, 9, 10, 11, 12 and 13, demonstrated that the fitted models provided a good overall representation of the measured data. The residuals for the anterior, posterior, and lateral orientations for each radionuclide were generally distributed around zero with no strong systematic trends or distance-dependent bias observed. While minor structured deviations were observed for each average-orientation dataset, no consistent increase or decrease in residuals with distance was found. The overall residual magnitudes remained low, indicating that the weighted fitting procedure provided a satisfactory representation of the measured data and adequately captured the underlying dose-rate behaviour.

The Gamma Factor (GF), also known as the Specific Gamma-Ray Dose Constant is defined as the unshielded gamma ray dose-equivalent rate at a specific distance from a point-source of unit activity and was determined by evaluating the point source curve formula for a specific radionuclide at both 0.3 m and 1 m. The GF is strictly a measure of exposure rate and not the total effective dose equivalent as specified in the US-NRC Regulatory Guide 8.39 [7], as it does not account for attenuation and scatter in the patient or exposed individual [8].

The Self-absorption Factor (SF) is a unitless coefficient that accounts for the reduction in radiation intensity caused by the radioactive source’s own volume and its surrounding medium. The SF was determined by inserting the attenuated dose rate (phantom source) in the numerator, and the unattenuated dose rate (point source) in the denominator of Eq. 2.

2 $$ SF = \frac{{Attenuated\; dose\; rate\; at \;x\; {\mathrm{cm}}}}{{Unattenuated \;dose \;rate \;at\; x\; {\mathrm{cm}}}} $$

The variation of the SF with distance was implicitly captured through the distribution of SF values calculated across the full 10–100 cm range. The reported SDs reflected the spread of the values and demonstrated that, although the SFs fluctuated with distance (increased and decreased within the evaluated range), no systematic distance-dependent trend was observed. This indicated that the SF remained sufficiently stable to be meaningfully generalised over clinically relevant distances. Notably, the magnitude of the SDs reported for the phantom-derived SFs were substantially smaller than those reported for patient measurements, supporting their suitability as representative correction factors.

The uncertainties associated with Eq. 2 are included in the SDs found in Tables 2 and 3, with the maximum uncertainty being the SD divided by the SF value. These uncertainties include those arising from positioning errors, the detector, the activity of the measured radionuclide, and day-to-day variabilities.

## Results

Results from this study include external exposure curves for various radionuclides commonly used in NM. The results from point source models, line source models, and phantom models are presented in this section.

### Point source

All point source measurements using Tc-99m, F-18, I-131, and Lu-177 are described in the following section.

**Fig. 3:**
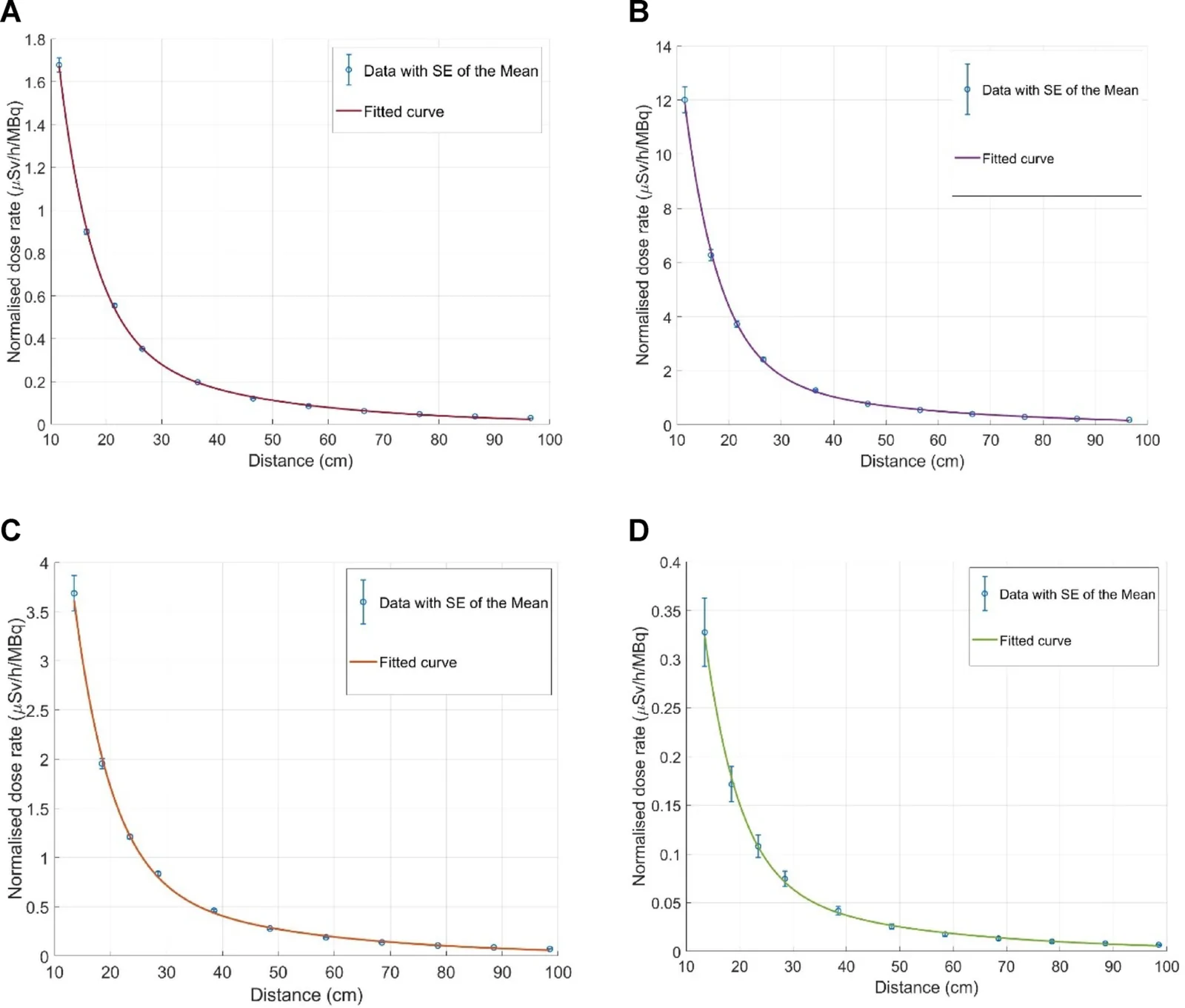
The plots show the relationship between the normalised dose rate (μSv/h/MBq) and distance (cm) for four radionuclides commonly used in NM. All plots include data points and the SE of the mean. Point source response curves are shown for four radionuclides: **A** Tc-99m, **B** F-18, **C** I-131, and **D** Lu-177. The parameters of the fitted curves are provided in Table 4

As expected, the normalised dose rate measurements presented in Fig. 3 show a decreasing trend as distance increases. These graphs highlight a rapid dose-falloff and demonstrate that by creating distance between the radioactive source and staff, other individuals are able to drastically reduce the amount of ionising radiation they are being exposed to.

GFs for the radionuclides shown in Fig. 3 are presented in Table 1. The GFs for each radionuclide are evaluated at both 0.3 m and 1.0 m. Percentage differences, were calculated by comparing these values with those reported in the literature.

**Table 1:** GFs (μSv/h/MBq) for various radionuclides based on the point source curves in Fig. 3

Radionuclide	Tc-99m	Tc-99m	F-18	F-18	I-131	I-131	Lu-177	Lu-177
Distance	0.3 m	1.0 m	0.3 m	1.0 m	0.3 m	1.0 m	0.3 m	1.0 m
NLM Fit	0.275	0.0239	1.84	0.161	0.721	0.0552	0.0636	0.00543
*Literature*								
Madsen et al. [20], Diff	–	–	–	0.143, + 13%	–	–	–	–
Delacroix et al. [17], Diff	0.261, + 5%	0.0224, + 7%	1.81, + 2%	0.158, + 2%	0.729, − 1%	0.0636, − 13%	–	–
Shleien [18], Diff	–	0.0332, − 28%	–	0.188, − 14%	–	0.0765, − 28%	–	0.00764, − 29%
CNSC [19], Diff	–	0.0185, + 29%	–	0.140, + 15%	–	0.0547, + 1%	–	0.00494, + 10%
Wasserman and Groenewald [23], Diff	–	–	–	0.149, + 8%	–	0.0561, − 2%	–	–
Hosono et al. [22], Diff	–	–	–	–	–	–	–	0.00517, + 5%

The curves are evaluated at both 0.3 m and 1.0 m and compared with values reported in the literature. The GF value defines the intensity of gamma or X-ray radiation produced by a specific radionuclide at a specified distance

Table 1 shows that the GFs derived in this study are generally consistent with published values, with each radionuclide and distance being within 10% of a literature value. The differences between the values obtained in this study and those reported in the literature mainly reflect variations in source modelling, dosimetric definitions, and experimental methodology. Larger discrepancies are observed at 1.0 m, indicating a dominant contribution from systematic effects such as photon scatter.

### Line source

All line source measurements using Tc-99m and F-18 are described in the following section.

**Fig. 4:**
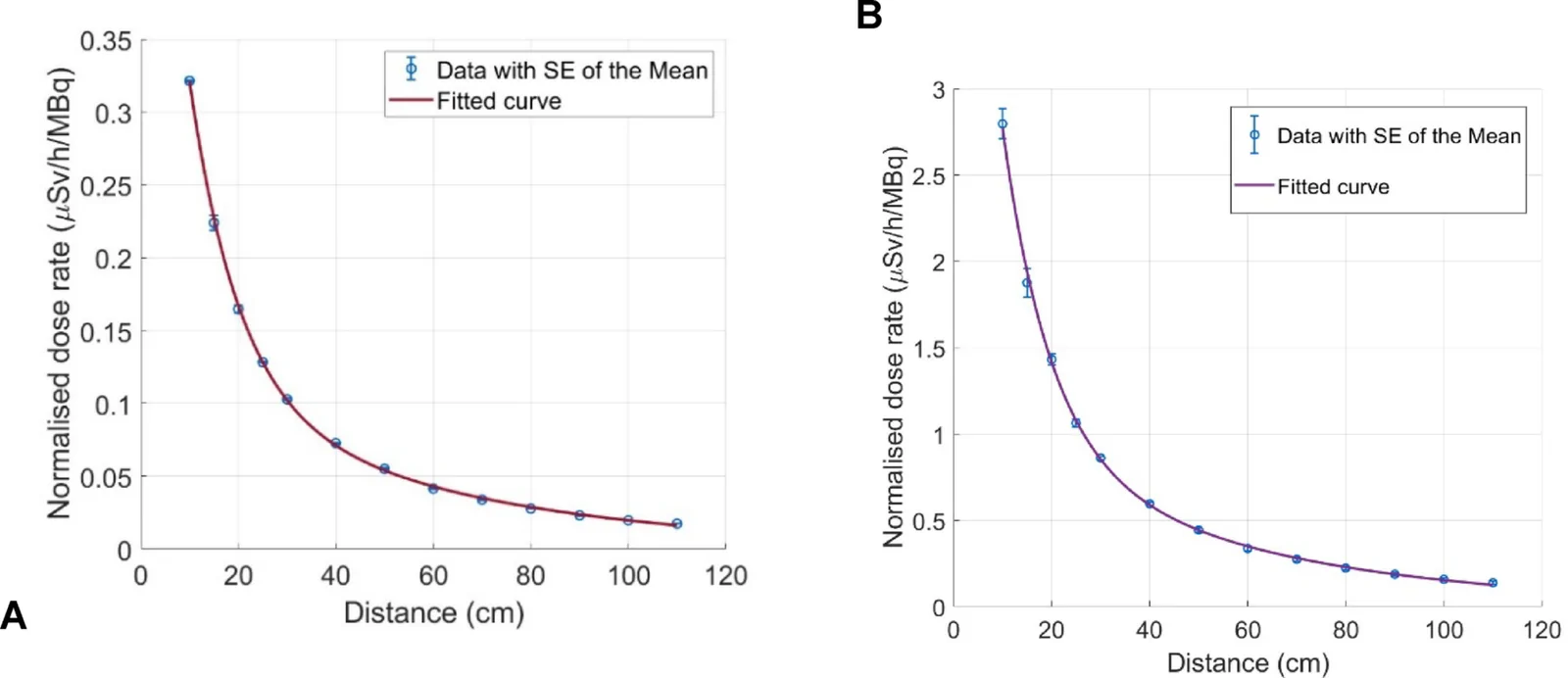
The plots show the relationship between the normalised dose rate (μSv/h/MBq) and distance (cm) for two radionuclides commonly used in diagnostic imaging. Data points and the SE of the mean shown in all plots. **A** Tc-99m line source curve. **B** F-18 line source curve. The parameters of the fitted curves (Eq. 1) for the line source measurements are listed in Table 4

For the line source geometry, both radionuclides exhibited a smooth, decrease in normalised dose rate with increasing distance. Tc-99m and F-18 displayed similar qualitative behaviour; however, F-18 consistently produced higher normalised dose rates across all distances, reflecting its higher photon energy and greater penetrability. Qualitatively, the line source curves were less steep than the respective point source curves, indicating that the normalised dose rate was decreasing at a slower rate (Fig. 4).

### Homogeneous radionuclide and water filled phantom

All NEMA phantom measurements using Tc-99m, F-18 and Lu-177 are described in the section below.

**Fig. 5:**
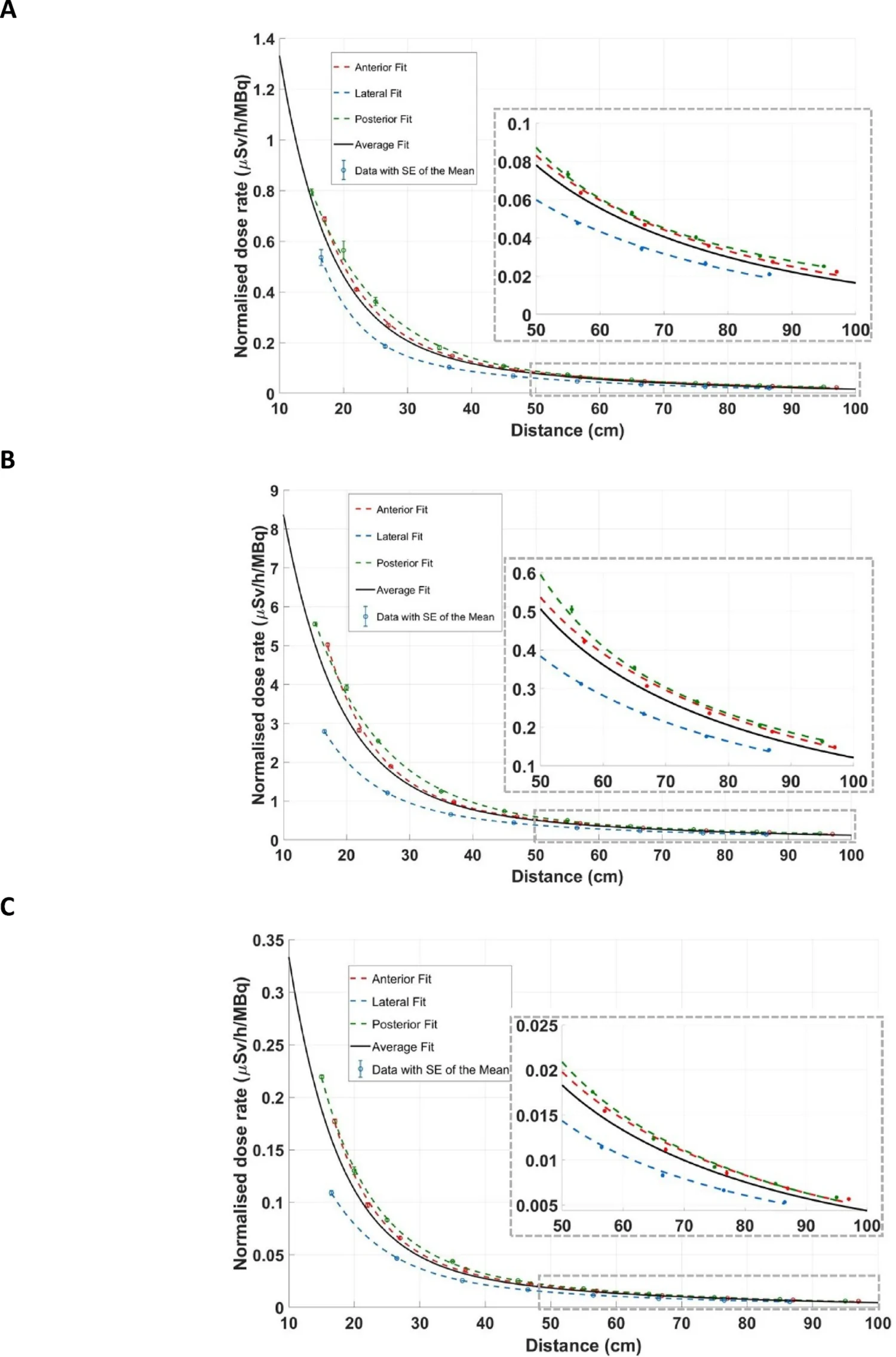
The plots show the relationship between the normalised dose rate (μSv/h/MBq) and distance (cm) for three different orientations of the NEMA phantom, as well as an average curve for three radionuclides. The three orientations were anterior, posterior, and lateral. All orientation plots show data points and the SE of the mean. Phantom source graphs for **A** Tc-99m, **B** F-18, and **C** Lu-177. The parameters of the fitted curves for the NEMA phantom measurements are provided in Table 4

**Fig. 6:**
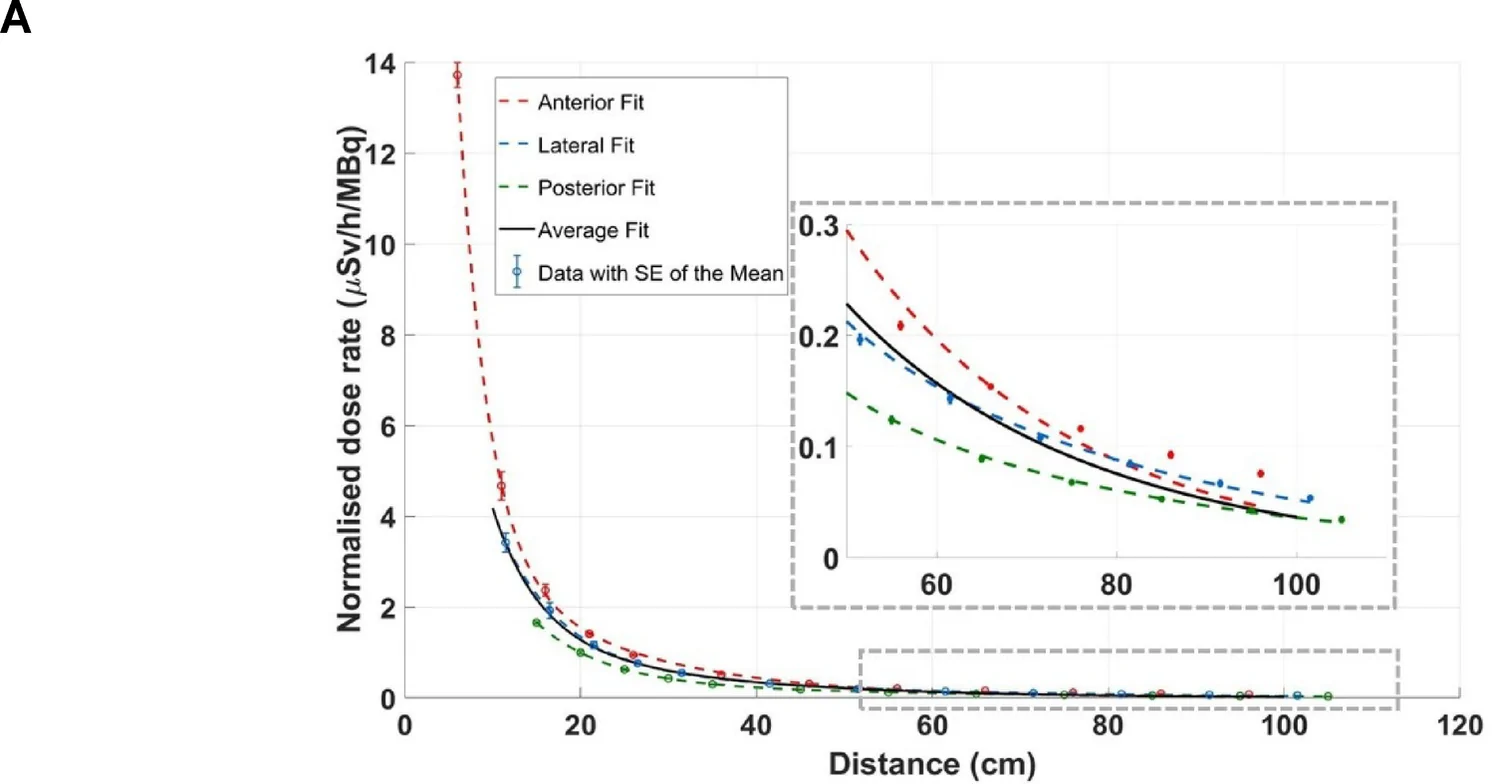
The plots show the relationship between the normalised dose rate (μSv/h/MBq) and distance (cm) for three different orientations of the neck phantom, as well as an average curve. All orientation plots display data points and the SE of the mean. The parameters of the fitted curves (Eq. 1) for the I-131 neck phantom measurements are provided in Table 4

**Fig. 7:**
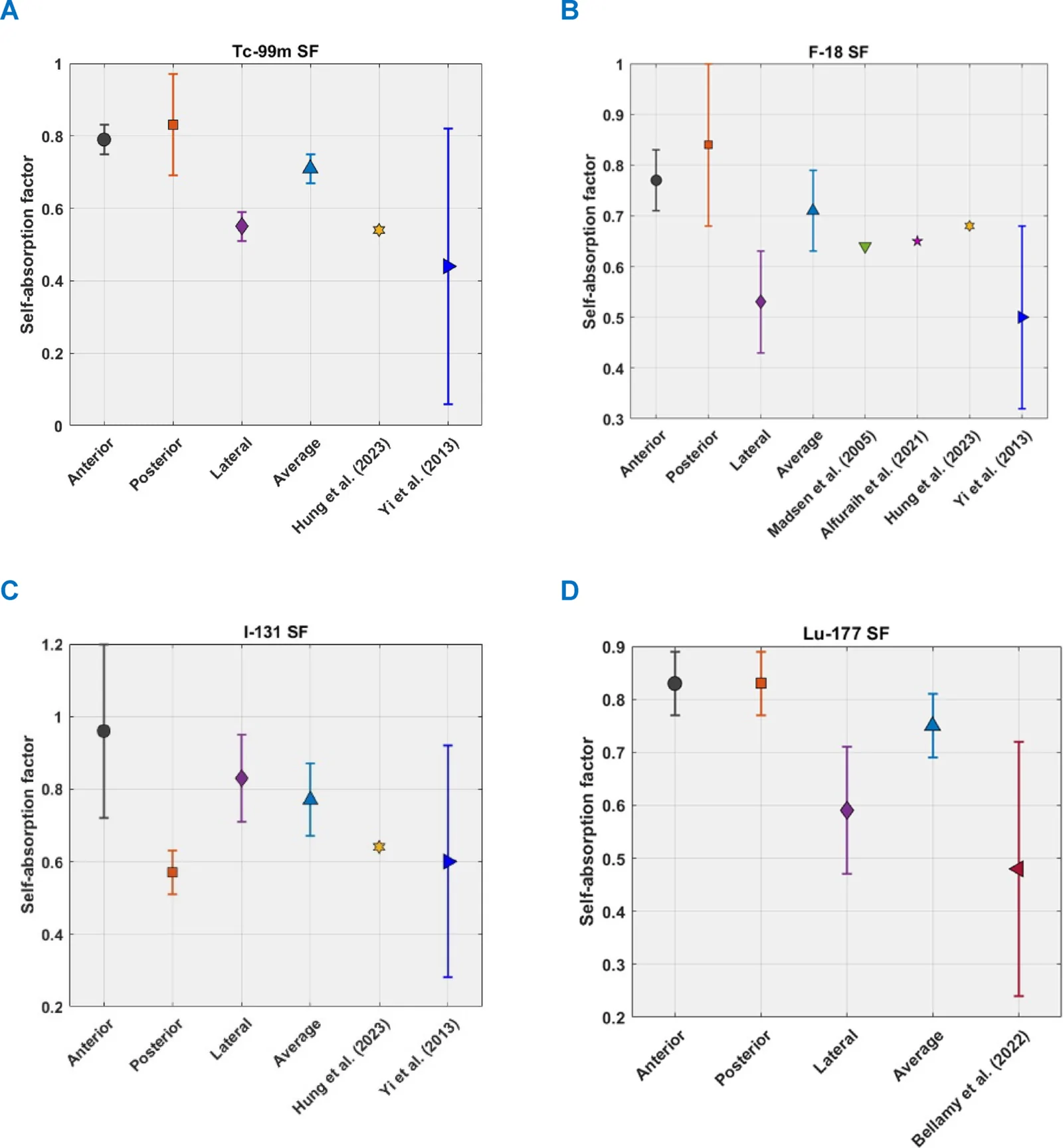
SF graphs for four radionuclides and the respective comparisons to literature. **A** Tc-99m, **B** F-18, **C** I-131, and **D** Lu-177. Error bars are two SDs

**Fig. 8:**
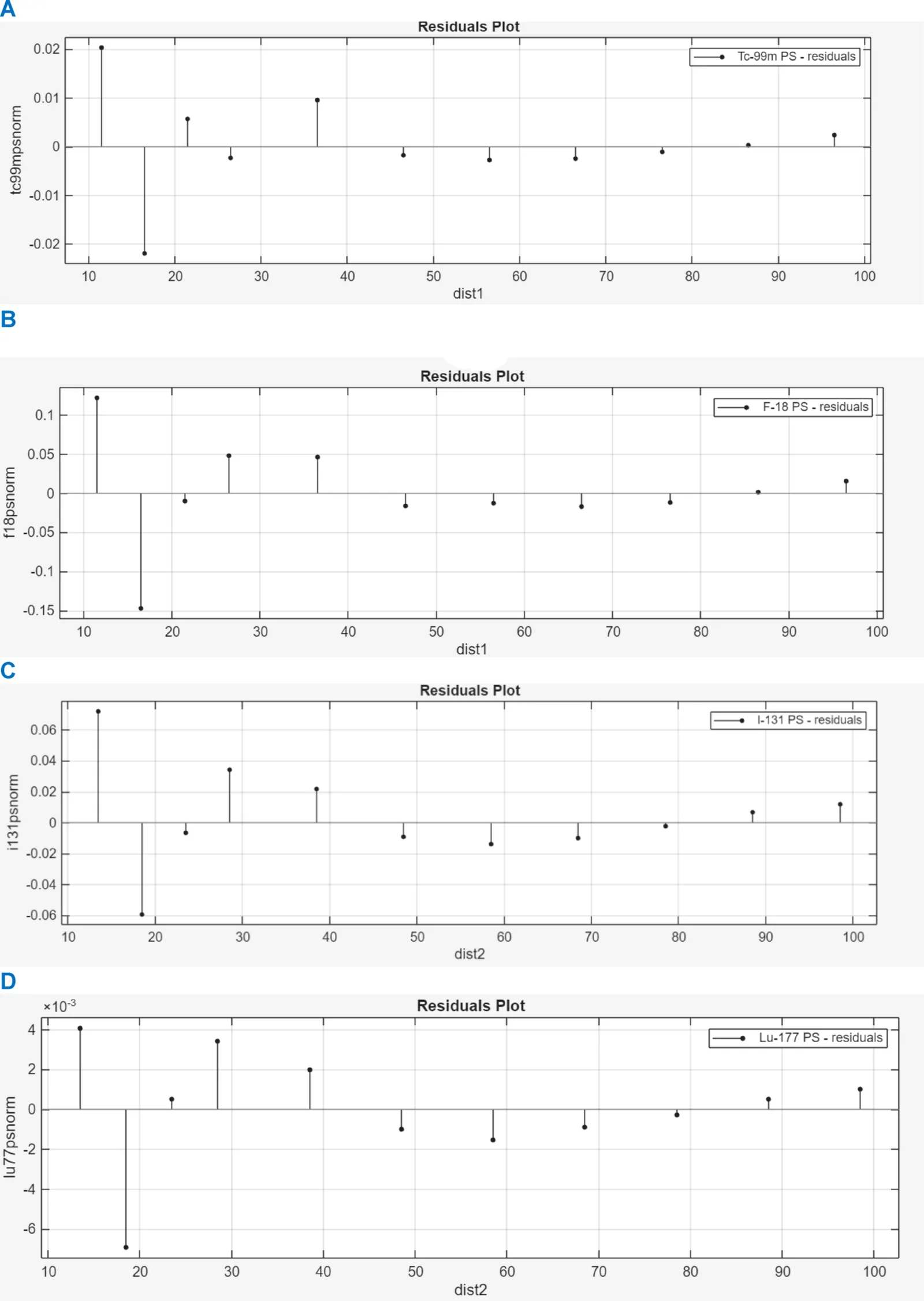
Residual plots for the four point source curves. **A** Tc-99m, **B** F-18, **C** I-131, and **D** Lu-177

**Fig. 9:**
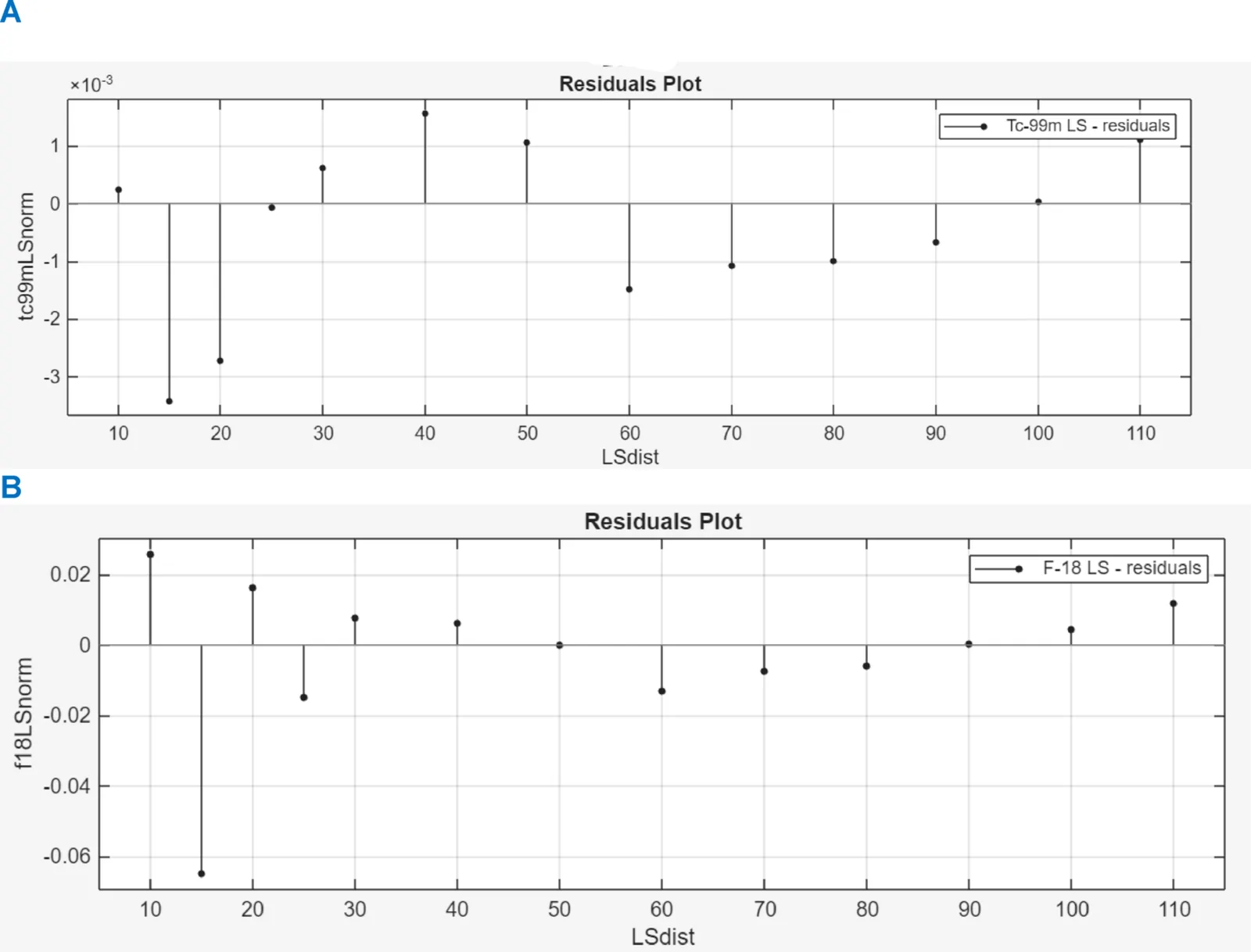
Residual plots for the two line source curves. **A** Tc-99m, **B** F-18

**Fig. 10:**
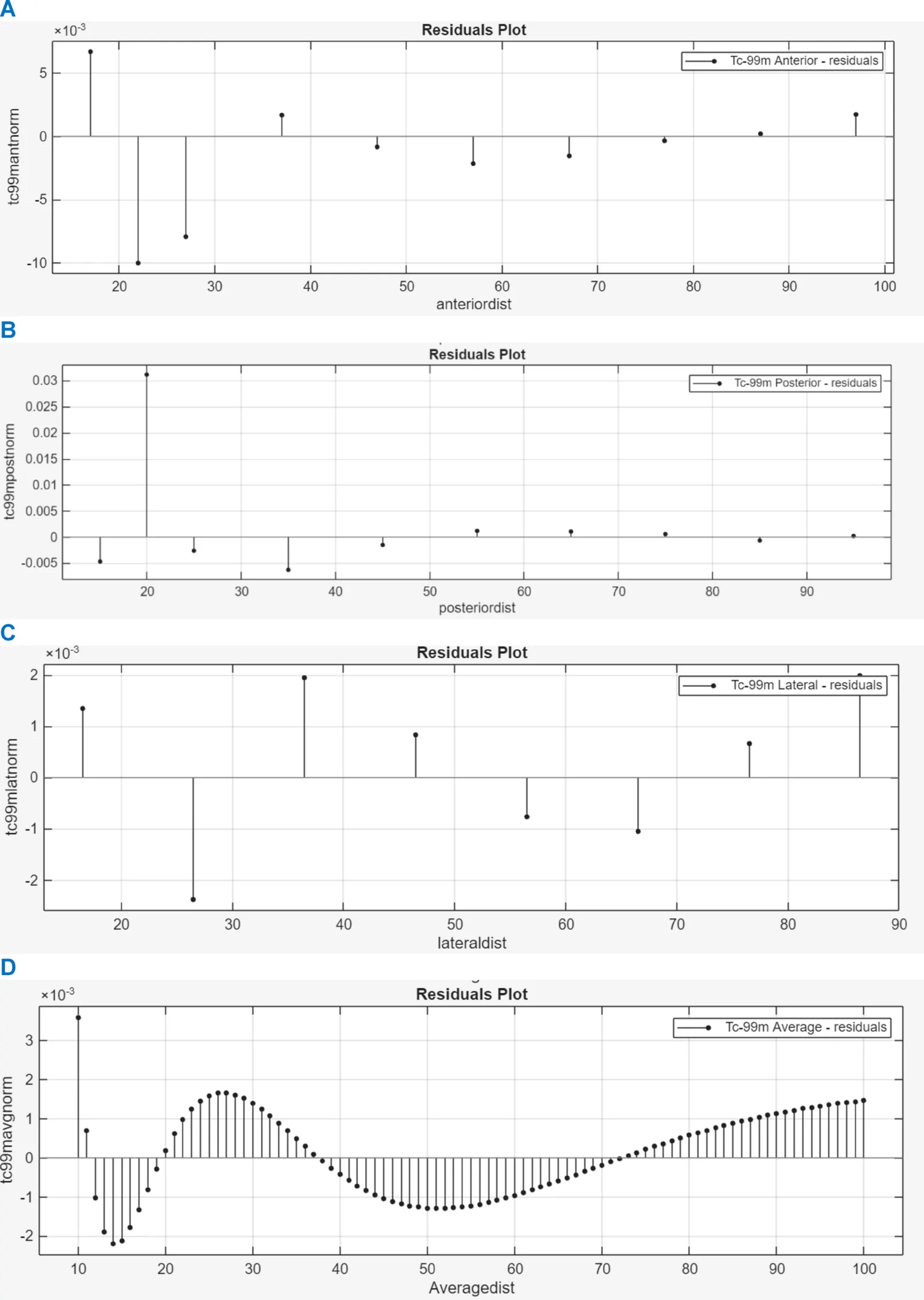
Residual plots for the Tc-99m phantom source curves. **A** Anterior, **B** Posterior, **C** Lateral, and **D** Average

**Fig. 11:**
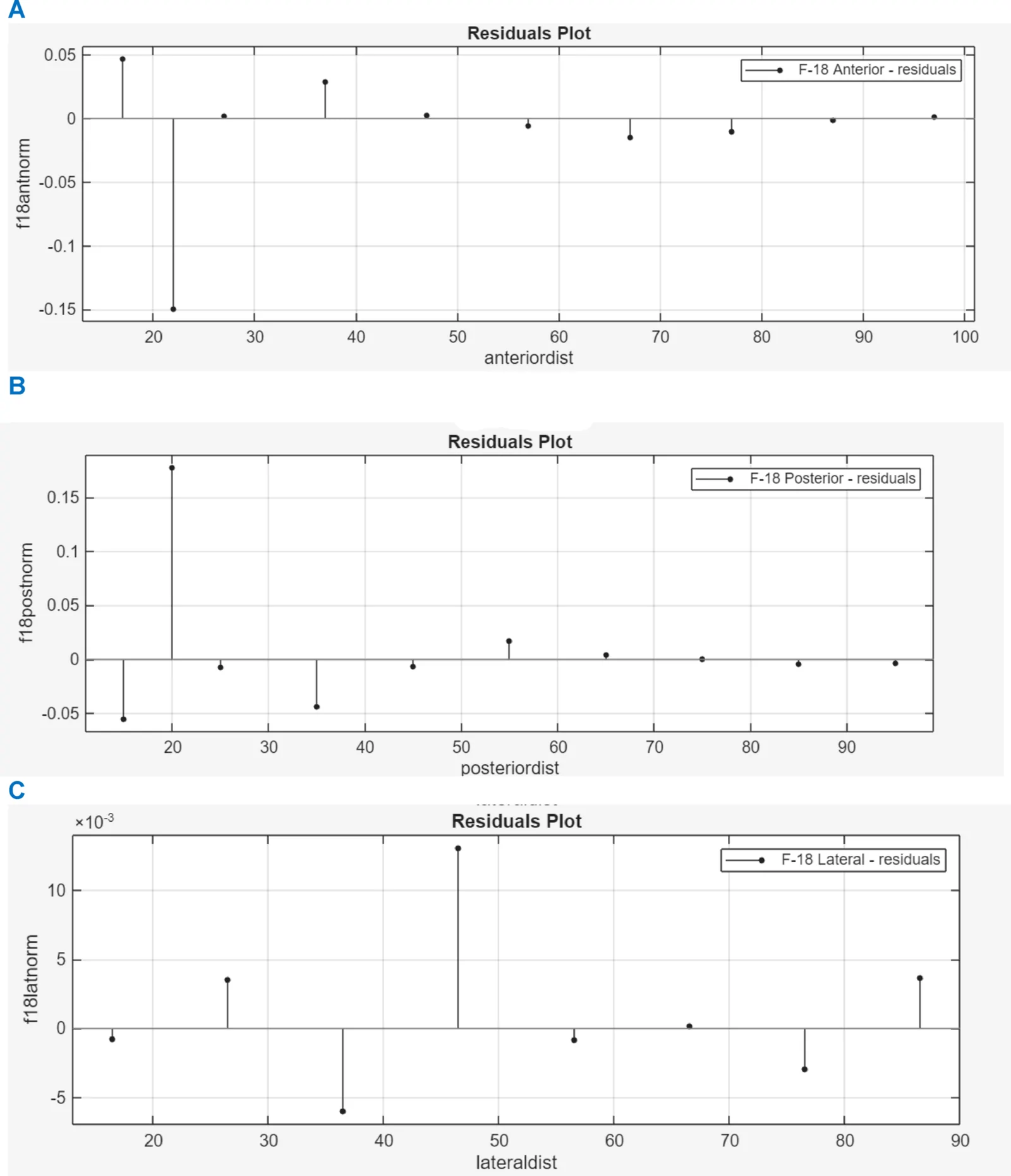

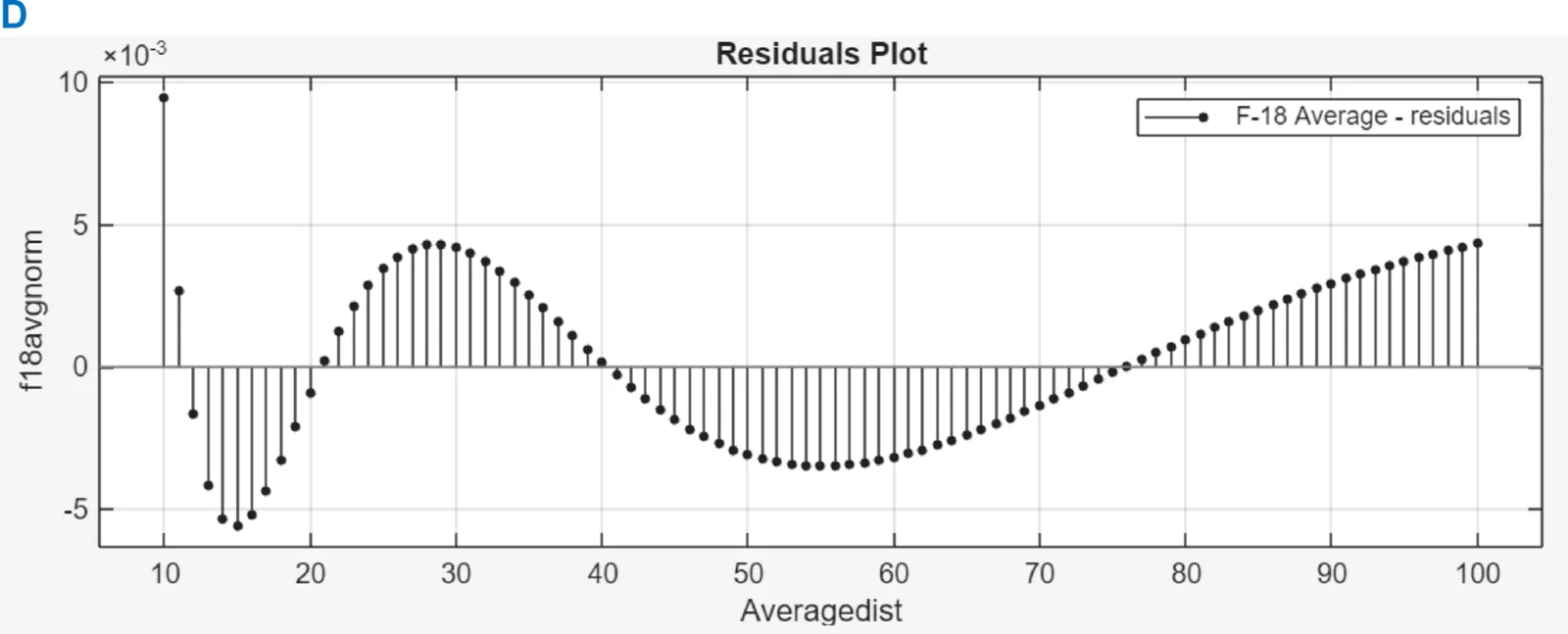
Residual plots for the F-18 phantom source curves. **A** Anterior, **B** Posterior, **C** Lateral, and **D** Average

**Fig. 12:**
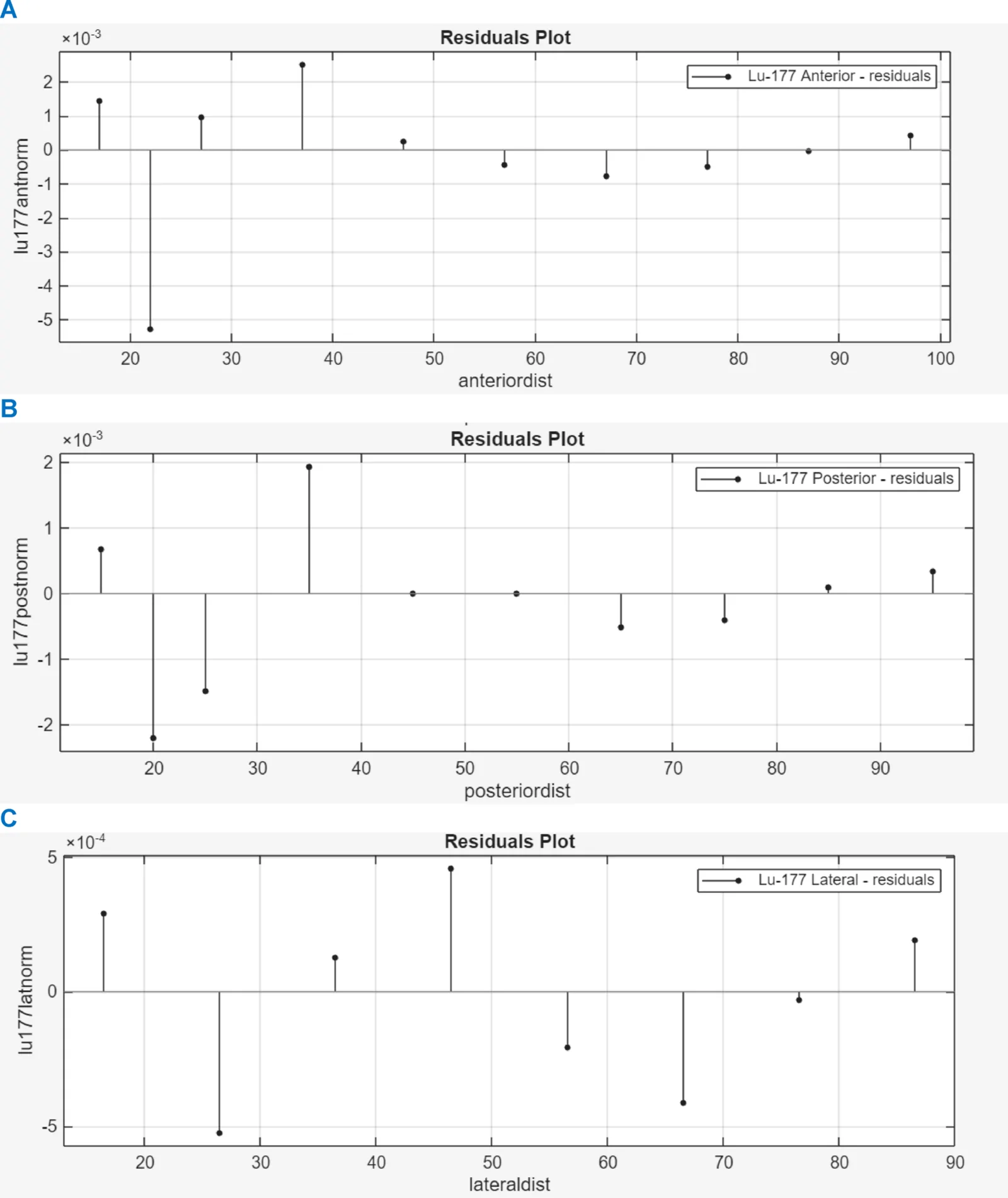

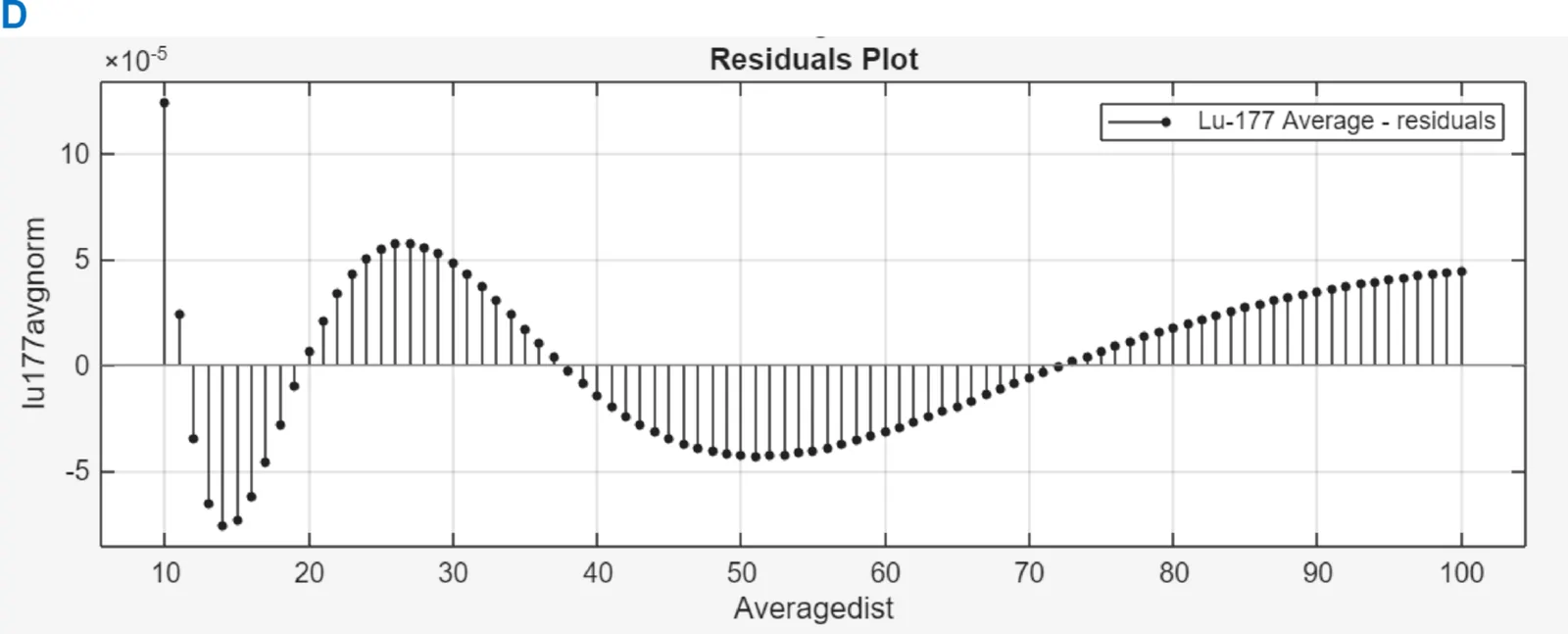
Residual plots for the Lu-177 phantom source curves. **A** Anterior, **B** Posterior, **C** Lateral, and **D** Average

**Fig. 13:**
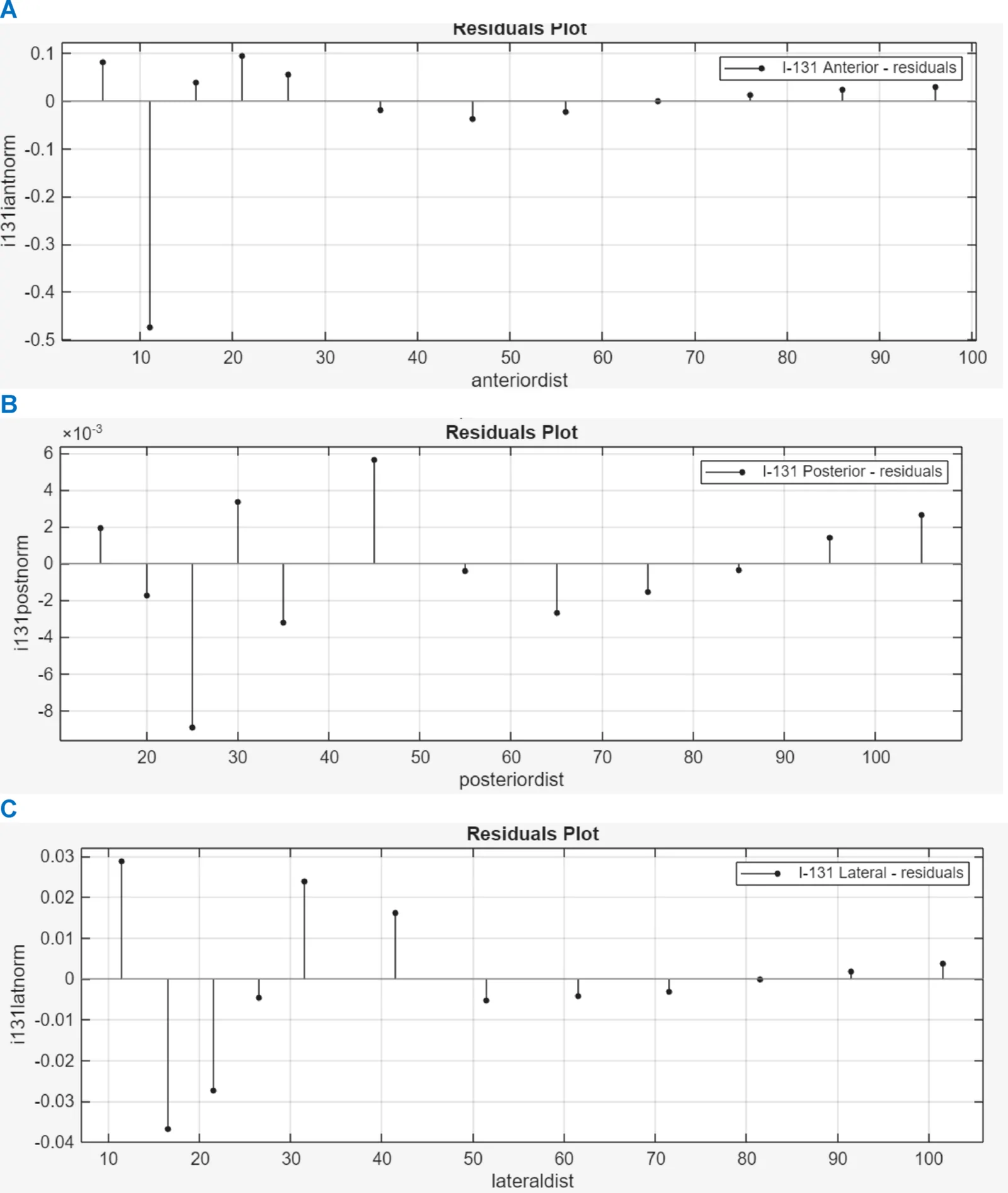

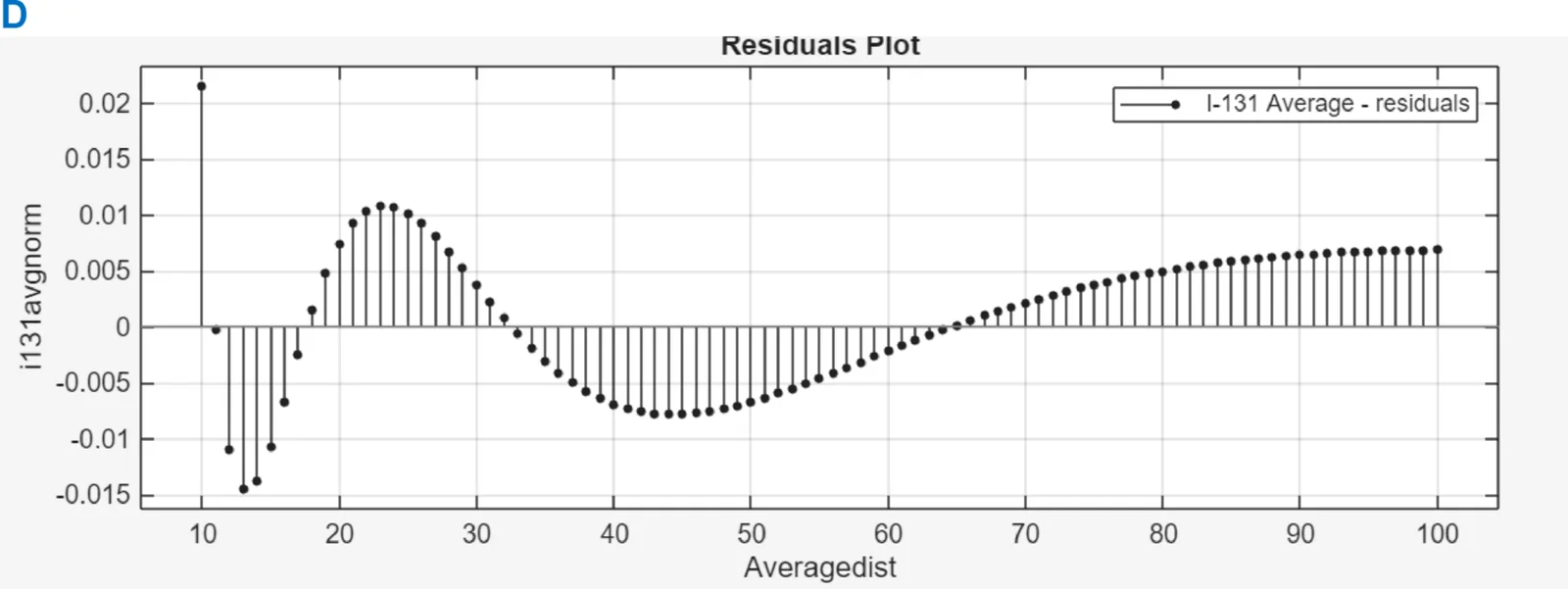
Residual plots for the I-131 phantom source curves. **A** Anterior, **B** Posterior, **C** Lateral, and **D** Average

SFs for the same radionuclides in Figs. 5 and 6 are shown in Tables 2 and 3, respectively. The values and comparisons made in Tables 2 and 3 are visualised in Fig. 7. The SFs for each orientation were calculated using Eq. 2. An average and SD were calculated from ninety-one SFs, covering distances from 10 to 100 cm, inclusively. The average phantom curve was obtained by evaluating each orientation from 10 to 100 cm and averaging at each distance.

**Table 2:** SFs calculated for various radionuclides using the NEMA phantom. Literature values are based on either patient measurements or simulations

Orientation	Tc-99m	F-18	Lu-177
*Measured self-absorption factor (SD)*			
Anterior	0.79 (0.02)	0.77 (0.03)	0.83 (0.03)
Posterior	0.83 (0.07)	0.84 (0.08)	0.83 (0.03)
Lateral	0.55 (0.02)	0.53 (0.05)	0.59 (0.06)
Average	0.71 (0.02)	0.71 (0.04)	0.75 (0.03)
*Literature self-absorption factor (SD)*			
Madsen et al. [20]	–	0.64	–
Alfuraih et al. [24]	–	0.65	–
Hung et al. [25]	0.54	0.68	–
Yi et al. [2]	0.44 (0.19)	0.50 (0.09)	–
Bellamy et al. [29]	–	–	0.48 (0.12)

Values are presented as the mean and SD in round brackets of the oriented phantoms divided by the corresponding point source curves, both evaluated from 10 to 100 cm. Physically, the SF value represents the ratio of reduced intensity of radiation compared to the unattenuated source, primarily due to internal absorption and attenuation from the NEMA phantom

**Table 3:** SFs calculated for I-131 using the neck phantom. Literature values are based on either patient measurements or simulations

Orientation	I-131
*Measured self-absorption factor (SD)*	
Anterior	0.96 (0.12)
Posterior	0.57 (0.03)
Lateral	0.83 (0.06)
Average	0.77 (0.05)
*Literature self-absorption factor (SD)*	
Hung et al. [25]	0.64
Yi et al. [2]	0.60 (0.16)

Values are presented as the mean and SD of the oriented phantom curve divided by the point source curve, both evaluated from 10 to 100 cm. Physically, the SF value represents the ratio of reduced intensity of radiation detected compared to the unattenuated source, primarily due to the internal absorption and attenuation from the neck phantom

**Table 4:** Parameters used for all calculated model- fitted curves, including point, line, and phantom models, following Eq. 1

Parameter	F	g	H	k
Figure 3				
A	7.152	0.145	0.435	0.029
B	55.258	0.150	2.933	0.029
C	21.366	0.150	1.225	0.031
D	2.020	0.155	0.109	0.030
Figure 4				
A	0.582	0.099	0.127	0.019
B	5.171	0.102	1.109	0.020
Figure 5				
A				
Anterior	4.614	0.132	0.315	0.028
Posterior	2.759	0.095	0.181	0.021
Lateral	6.257	0.172	0.275	0.031
Average	4.190	0.135	0.324	0.030
B				
Anterior	36.973	0.133	1.760	0.026
Posterior	19.492	0.094	1.196	0.021
Lateral	15.503	0.126	1.313	0.026
Average	23.275	0.119	1.633	0.026
C				
Anterior	1.572	0.147	0.073	0.027
Posterior	1.161	0.129	0.075	0.028
Lateral	0.569	0.120	0.046	0.025
Average	1.089	0.135	0.066	0.027
Figure 6				
A				
Anterior	48.004	0.233	2.229	0.040
Posterior	8.580	0.124	0.479	0.026
Lateral	12.692	0.130	0.719	0.026
Average	18.837	0.173	1.391	0.036

Similarly to the point source curves in Fig. 3, the normalised dose rate measurements from the NEMA phantoms presented in Fig. 5 showed a decreasing trend as distance increased. The interval ranges for the graphs in Fig. 5 were narrower than the respective point source curves in Fig. 3, demonstrating the impact of additional attenuation from the phantom on the amount of radiation being detected.

The lateral SFs for the radionuclides measured in the NEMA phantom, as seen in Fig. 5 and Table 2, were smaller than the other measured orientations. This orientation exhibited greater attenuation as the phantom was non-circular, rather, it was an ellipse, as seen in Fig. 1. The maximum uncertainty associated with the SF values using the NEMA phantom was 9.5%, calculated by dividing the maximum SD (0.08) by the F-18 SF for the posterior orientation (0.84), as mentioned in the Materials and Methods section.

### Neck phantom

All neck phantom measurements using I-131 are described in this section.

Similarly to the point source curves in Fig. 3, the normalised dose rate measurements from the neck phantom presented in Fig. 6 showed a decreasing trend as distance increased. The interval ranges for the graphs in Fig. 6 were narrower than the respective point source curve for I-131 in Fig. 3, demonstrating the impact of additional attenuation from the phantom on the amount of radiation being detected.The shape of the curves in Fig. 6 are different than those of Fig. 5, highlighting the differences in phantom source geometry.

The posterior SF for I-131 measured in the neck phantom, as seen in Fig. 6 and Table 3, was smaller than the other measured orientations. This orientation exhibited the greatest attenuation as the source was located closer to the anterior orientation than the posterior orientation, as seen in Fig. 1. The maximum uncertainty associated with the SF values using the neck phantom was 12.5%, calculated by dividing the maximum SD (0.12) by the I-131 SF for the anterior orientation (0.96), as mentioned in the Materials and Methods section.

The SF values from Tables 2 and 3 are visualised in Fig. 7 to show how the findings of this study using geometry-specific homogenous water phantoms compare with published results from Monte Carlo simulations and patient measurements.

## Discussion

An evaluation and comparison of the values measured in this study with those reported in the literature are described below. As expected, the dose rate per unit activity decreases with increasing distance for all measurements, including point, line, and phantom source models.

The normalised dose rate curves are distributed over a wide interval for F-18 and I-131, while those for Tc-99m and Lu-177 they are confined to a narrower interval. This behaviour was attributed to both the photon energies emitted by each radionuclide and the dependence of the measured dose rate on source-to-detector distance. At shorter distances (10–30 cm), the change in distance produces a more pronounced effect on the measured dose rate, resulting in greater separation between successive data points. As the distance increases, the change in dose rate between consecutive measurement positions becomes progressively smaller, causing the data points to become less dispersed. The observed decrease in dispersion with increasing distance originates from the geometric spreading of radiation as it propagates away from the source. F-18 and I-131 emit higher-energy photons than Tc-99m and Lu-177, leading to higher normalised dose rates across the measurement range, and resulting in a larger absolute reduction in dose rate with increasing distance, and therefore a wider interval of values. Consequently, the interval width decreases in the order of F-18, I-131, Tc-99m, and Lu-177.

### Point source

The GF results evaluated at 0.3 m and 1.0 m from the point source curves, for various radionuclides, are found in Table 1.

The GFs in this study at 0.3 m and 1.0 m for Tc-99m, F-18, and I-131 agree with the *Radionuclide and Radiation Protection Data Handbook,* Delacroix et al. [17], with five values being within 10%. The values in this study for Tc-99m and F-18 are slightly higher than the values published in the handbook. In this handbook, the external exposure from gammas and x-rays (deep tissue dose) was calculated using Microshield Version 4.10 (Grove Engineering, 15,215 Shady Grove Road, Rockville, USA, 1996). An unattenuated point source in air at a distance of 0.3 m, and a ‘penicillin’ type, 10 ml glass vial at a reference distance of 1.0 m in air were considered.

The GFs at 1.0 m for all radionuclides were in between *The Health Physics and Radiological Health Handbook,* Shleien [18] and *Radionuclide Information Booklet,* Canadian Nuclear Safety Commission [19], with the former publishing significantly higher values, especially for Tc-99m, I-131, and Lu-177, which led to differences of up to 29%. The reason for these higher values is explained in *AAPM Task Group 108: PET and PET/CT Shielding Requirements*, Madsen et al. [20], for the case of F-18. These values are calculated for the maximum dose received in a 30 cm slab of tissue from a beam of 511 keV annihilation photons, incorporating additional exposure from side and backscatter. The values from the *Radionuclide Information Booklet,* Canadian Nuclear Safety Commission [19] were calculated using fluence-to-effective dose conversion coefficients from the *International Commission on Radiological Protection’s (ICRP) Publication 116* [21]. The differences in agreement with this study were most prominent for Tc-99m and F-18, 29% and 15%, respectively, while there was closer agreement for Lu-177 and I-131, 10% and 1%, respectively.

There was also strong agreement for Lu-177 with Hosono et al. [22] at 1.0 m (5%), and I-131 with Wasserman and Groenewald [23] at 1.0 m (1%).

### Line source

Limited information on line source dose rates was found in the literature. No data were available for direct quantitative comparison with the results of this study. SFs were not calculated using the line source, as analysis of the ratio between the line source and phantom measurements demonstrated a consistent and systematic dependence on distance for both Tc-99m and F-18. In particular, the ratios of line source values to point source values increased with distance over the 1–100 cm range, consistent with the findings of Siegel et al. [8] for a line source of similar source length.

At shorter distances, the line source model was observed to be less conservative than the point source and phantom source, reflecting the impact of elongated source geometry. At larger distances, the line source model approached the point source model and exceeded the phantom model. The F-18 line source model approached the point source model faster than Tc-99m due to the higher energy of its emissions and reduced attenuation, exhibiting more point-source-like behaviour at distance.

The experimentally determined line source measurements presented here provide a useful intermediate representation between idealised point source models and phantom models. The line source geometry captures key features of extended activity distributions encountered in NM patients, providing a more representative approximation than the point source model for widespread activity distributions. These data offer a reproducible basis for future studies investigating line source geometries in NM dosimetry.

### Homogeneous radionuclide and water filled phantom

The SF results calculated from 10 to 100 cm using the point source and phantom source curves, for Tc-99m, F-18, and Lu-177, are found in Table 2, and visualised in Fig. 7.

The average SF for F-18, which accounts for contact with a NM patient with unknown geometry, agrees with the literature [20, 24, 25]. The values published in the literature were from MCNPX Monte Carlo simulation codes [26, 27] using the Zubal voxel phantom [28] and Oak Ridge National Laboratory (ORNL) adult phantoms, with SFs being 0.65 and 0.68, respectively [24, 25]. The latter SF falls within one SD of the average phantom SF calculated in this study (0.71, 0.04).

The SF for F-18 in the lateral orientation (0.53, 0.05), agrees with patient measurements (0.50, 0.09) made by Yi et al. [2]. When considering one SD, the corresponding uncertainty intervals [0.48, 0.58] and [0.41, 0.59] exhibit overlap, as seen in Fig. 7, indicating that the two measurements are consistent within their respective experimental uncertainties.

The SF for Tc-99m in the lateral orientation (0.55, 0.02), agrees with simulations (0.54) performed by Hung et al. [25]. The lateral (0.55), anterior (0.79) and average (0.71) SF values all lie within two SDs from the values (0.44, 0.19) reported by Yi et al. [2], as seen in Fig. 7, particularly due to the significantly large SD (0.19) which demonstrates high levels of patient variability.

The lateral orientation of the NEMA phantom has the most attenuation compared with the anterior and posterior orientations, highlighting how additional attenuation decreases external exposure. The reason the SFs in posterior and anterior orientations for Tc-99m, F-18, and Lu-177 were larger in this study compared with the literature was twofold. Firstly, the complexity and variability of patient anatomy was not encompassed in the simplicity of the phantom, such as bone, soft tissue, and organs, which affects the amount of radiation that is absorbed, e.g. relatively higher photoelectric absorption in higher attenuating tissue. Secondly, the phantom depicts the abdomen of the patient, not the entirety of the patient. It is likely that the additional attenuation from the centre of the phantom in the lateral orientation counteracted these effects.

The SFs for Tc-99m, F-18, and Lu-177 in this study were comparable for the same orientations. This was not expected, as it was assumed the lower-energy photons from Tc-99m and Lu-177 would be more attenuated than the higher-energy photons from F-18. This interesting result was likely due to the calculation of SFs from Eq. 2, as both the numerator and denominator were lower for Tc-99m and Lu-177. This led to the ratio between attenuated and unattenuated dose rates in water being remarkably similar for all radionuclides.

The Lu-177 SF for the lateral orientation lies within one SD of the value published by Bellamy et al. [29]. The GF used in their study to compute the SF was substantially higher than the 95th percentile of patient measurements and was found in *The Health Physics and Radiological Health Handbook* [18]. The study claimed that, in the absence of patient measurements, a suitable lower value could be used, such as the value obtained in this study (0.00543 μSv/h/MBq).

### Neck phantom

The SF results calculated from 10 to 100 cm using the point source and phantom source curves for I-131 are found in Table 3.

The SF for the anterior orientation (0.96, 0.12) was close to unity, which was expected due to minimal attenuation between the detector and the source. This value skewed the average SF, causing it to be substantially higher than the literature. If the anterior SF value was ignored, the average SF value would be closer in agreement with the literature. The value (0.64) published by Hung et al. [25] lies between the posterior (0.57) and average (0.77) SF values. The posterior SF (0.57, 0.03) of the neck phantom was the orientation with the most attenuation, and there was strong agreement with patient measurements (0.60, 0.16) made by Yi et al. [2]. The lateral (0.83), posterior (0.57) and average (0.77) SF values all lie within two SDs of the SF value (0.60, 0.16) reported by Yi et al. [2], as seen in Fig. 7. Similarly to the Tc-99m findings, the patient measurements for I-131 also had a high SD (0.16).

Analysis of the results indicated that the measured point-source model overestimates the dose rate by a factor of 1.45 at 1.0 m when compared to the average neck phantom model. While this finding aligns with the general departure from point-source assumptions identified in prior literature, the specific magnitude of the ratio reflects the localised focus of the phantom and omission of biokinetic modelling, which impacts external exposure estimates. As pre-defined exposure scenarios were not modelled in the present study, the average neck phantom model was used to compare with other findings. The average neck phantom model incorporates source geometry and attenuation from anterior, posterior, and lateral components, providing a baseline reference value, independent of orientation.

Dewji et al. [12] reported a higher benchmark overestimation of 1.75 ± 0.06 at 0.25 h post-administration using PIMAL (Phantom with Movable Arms and Legs) that integrated articulated whole-body geometry with complex biokinetic modelling, including time-dependent thyroid uptake and voiding patterns. Similarly, Han et al. [30] observed overestimation factors of approximately 1.6 for hyperthyroidism patients, reaching as high as 2.2 at 1.0 m for differentiated thyroid cancer (DTC) patients [31], by employing the UF/NCI hybrid phantom series. Closer agreement between the hyperthyroidism patients (1.6) and the average neck phantom (1.45) was physically expected due to the higher uptake (80%) of I-131 concentrated in the thyroid gland for the simulations, compared with the entire I-131 activity being placed in the cut-out of the neck phantom.

The lower ratio of 1.45 observed in the present study is likely attributable to the use of a neck phantom, which isolates the attenuation effects of the thyroid region without accounting for the additional volumetric shielding and scatter provided by the full-body anatomy present in the prior literature. Consequently, while the results confirm the conservative nature of point-source assumptions established by Han et al. [30] and Dewji et al. [12, 31], they provide a refined baseline specifically for thyroid-centric geometries, offering a distinct data point that complements the broader whole-body simulations of previous works.

### Clinical significance

The point-source results were in excellent agreement with internationally recognised values in the literature, demonstrating that they can be experimentally reproduced to a high degree of accuracy using a simple, cost-effective set-up.

Having dose rate measurement data for different source models and different radionuclides across the entire clinical range is highly valuable, for contextualising external radiation exposure scenarios in NM. The findings of this study are not intended to represent patient-specific corrections, nor to replace anatomically realistic phantoms or patient-based dosimetry models. Rather, they are provided as geometry-specific, conservative reference values that complement existing dose-assessment approaches and reduce reliance on unattenuated point-source assumptions, particularly at short distances where such assumptions are known to overestimate exposure.

The main advantage of having this data lies in supporting qualitative and comparative assessments, such as understanding relative changes in external dose rate with distance, orientation, and source geometry under reproducible experimental conditions. For example, the combined use of the measured GFs from Table 1 multiplied by SFs from Tables 2 and 3 for the specific radionuclide and subsequently corrected for the distance between occupational worker and radioactive NM patient by using the NLM fit from Eq. 1 allows relative comparisons between source models and radionuclides and illustrates how deviations from inverse-square behaviour emerge in clinically relevant near-field geometries. These results provide empirical context to support informed discussion of radiation protection considerations, such as scheduling considerations, shielding assessments, procedure planning, and proximity-dependent exposure without implying formal integration into structured dose decision tools.

In unexpected clinical situations, such as diagnostic radiology, ultrasound imaging, or emergency surgical procedures involving recently administered NM patients, the model proposed by this study provides a tool which may support informed decision making for radiation protection professionals to advise clinicians the external exposure to staff; for example, if aforementioned unexpected clinical situations with radioactive NM patients need to be delayed and for how long.

Structural radiation shielding in NM departments and Theranostics clinics is a delicate balance between appropriate shielding of surrounding clinical areas from radioactive patients, and structural costs. The required barrier thickness is a function of a variety of variables but essentially is dependent on the chosen GF and SF. The study showed that there is a wide range in GFs and SFs which can potentially lead to over- and under-shielding of such departments. The GFs and SFs provided by this study were measured in a controlled environment with high rigour.

Moreover, this data provides experimentally grounded reference data that enhances understanding of distance-dependent and geometry-dependent external dose behaviour in NM. The results are intended to complement—not replace—existing conservative dose estimation methods and to support comparative evaluations and contextual decision-making within established radiation protection frameworks, rather than serving as prescriptive or patient-specific dose guidance.

### Limitations

A notable limitation of this study was the inability to calculate SFs using the line-source model as the unattenuated source in the same way as was done with the point source. The field-of-view limitation means that, at closer distances, the dose rate was underestimated, as the detector coverage angle was only able to ‘see’ a fraction of the line source length.

Phantom measurements, and consequently SFs, are not directly comparable with patient measurements and simulations as previously alluded to, due to the non-uniformity of the radiation field surrounding a patient, and the phantoms in this study representing simplistic cross-sections of human anatomy, not the entire patient anatomy. Tissue heterogeneity, organ-specific activity distributions, and skeletal attenuation present in real patients were not represented. It is also worth noting that the phantoms used may underestimate variability due to differences in body habitus, as posture and habitus were not evaluated. All measurements were performed using static source distributions, and time-dependent redistribution, uptake, and clearance of radiopharmaceuticals—known to influence external dose rates—were not modelled. No validation against patient measurements or Monte Carlo simulations was performed as part of this study.

Another limitation was the precision of measurement of source-detector distance. The recorded dose rate was sensitive to small shifts in the position of the detector or source; this was more significant for the measurements closest to the source. While waiting for the reading on the detector to stabilise, the exact location of the ionisation chamber had the potential to shift by up to a few millimetres, as outlined in the uncertainty section.

### Uncertainty

The average error associated with the dose rate calibrations for the detectors was -2.91%. The accuracy of the detector was ± 10%, and the precision within 10% [32].

The electrometer accuracy for all the dose calibrators was less than ± 2%, with system precision less than ± 0.1% of full-scale deflection and system linearity within ± 2%.

The energy dependence for both Tc-99m and Lu-177 was underestimated by < 5%, with I-131 underestimated by < 2%, and negligible underestimation for F-18 according to Fluke Biomedical [32].

The uncertainty in the marking of distances was ± 3 mm.

### Future directions

Future studies should focus on expanding the dataset to include additional radionuclides and patient geometries. More contemporary and recently studied Theranostics radionuclides would benefit from further investigation, such as Tb-161, Zr-89 and Ac-225, albeit the latter being an alpha-emitter but yet having low abundant gamma emissions in the decay chain. Extension of this work to patient-specific dose estimation would require validation with anatomically realistic phantoms, in vivo measurements, or Monte Carlo modelling incorporating patient variability, which is beyond the scope of the present study.

Importantly, the development of a user-friendly software tool that incorporates these experimentally derived models could support rapid, conservative estimation of external dose-rate trends in clinical environments. Such a tool would be intended to assist radiation safety officers and clinicians by providing contextual and comparative information consistent with established radiation protection principles, rather than enabling patient-specific dose calculation or real-time clinical decision thresholds.

Patient-specific factors such as body mass index (BMI), organ size, and sex have a considerable impact on a patient’s dose rate. Bellamy et al. [29] found that there is a statistically significant inverse relationship between patient dose rate and patient BMI, an expected finding due to lower-BMI patients generally having less self-shielding. A future study that investigates how different patient-specific factors such as body habitus, tissue heterogeneity, and biokinetic redistribution influences self-absorption and external exposure would provide a tailored modelling framework that considers patient variability, which is an important direction for future work.

Monte Carlo simulation is an incredibly useful tool that could address patient variability; this approach was utilised by Hung et al. [25] to demonstrate that, when evaluating patient self-absorption, a reference phantom that best represents the people of a country or region should be selected in order to account for variability in physical characteristics of patients.

## Conclusion

Healthcare providers that work in close proximity to NM patients are exposed to higher levels of ionising radiation, raising concerns among staff members [2]. This study provides additional empirical dose rate measurements for commonly used radiopharmaceuticals in NM, across clinically relevant distances, to support and refine existing dose-assessment approaches. The measured data may assist in informing decisions regarding the timing of medical procedures involving NM patients, with the aim of reducing occupational exposure where practicable.

A simple, inexpensive, and reproducible experimental setup was designed to measure dose rates at clinical distances using point source, line source, and phantom source models. The GF results from this study were consistent with internationally recognised results, with observed differences attributable to variations in experimental methodology. The SF were slightly higher than values reported in the literature, which is reasonable given differences in phantom composition, geometry, and attenuation characteristics.

The measured and modelled datasets presented in this work provide practical reference values for comparing unshielded and attenuated sources for various orientations and geometries commonly encountered in NM environments. By modelling the measured dose rate behaviour directly, reliance on overly conservative inverse square law scaling may be reduced within the studied distance range. These results may also be integrated into existing radiation safety assessments and software tools to complement established frameworks and support more realistic evaluations of occupational exposure.

## Data Availability

No datasets were generated or analysed during the current study.

## Appendix

See Figs. ^8^, ^9^, 10, 11, 12 and 13.
